# Plastid genome sequencing, identification of nuclear SNP markers, and quality assessment of medicinal rhizomatous herb *Polygonatum odoratum* (Asparagaceae) cultivars

**DOI:** 10.1002/ece3.7599

**Published:** 2021-05-02

**Authors:** Shiou Yih Lee, Zhihui Chen, Zhiming Chen, Jingrui Chen, Xinjian Zhang, Jiawen Pan, Qiang Fan, Wenbo Liao

**Affiliations:** ^1^ State Key Laboratory of Biocontrol and Guangdong Provincial Key Laboratory of Plant Resources School of Life Sciences Sun Yat‐sen University Guangzhou China; ^2^ Guangdong National Nature Reserve Administration Shaoguan China

**Keywords:** Chinese Pharmacopoeia, hybridization, Polygonati Odorati Rhizoma, RAD‐Seq, Solomon's seal, traditional Chinese medicine

## Abstract

*Polygonatum odoratum* (Mill.) Druce (Asparagaceae, Asparagales) is a widely cultivated medicinal herb in China. However, this useful herb is understudied despite being known as a medicinal resource with top grade medical and edible properties since long. In this study, *P. odoratum* and four cultivars were investigated. The variations in morphological characteristics and vegetative phases of each cultivar were observed. For genetic aspect, the plastid genome of *P. odoratum* varies in length from 154,569 bp to 155,491 bp, containing a large single‐copy region of 83,486–84,459 bp, a small single‐copy region of 18,292–18,471 bp, and two inverted repeats of 26,302–26,370 bp. A total of 131 genes were predicted, including 85 protein‐coding, 38 tRNA, and eight rRNA genes. Genome comparisons revealed a slight variation in the sequence across the five accessions, but two highly variable regions (*trn*C‐*pet*N and *rpl*32‐*trn*L) were detected when comparing the four different cultivars. For the RAD‐seq markers, a total of 33.64 Gb of clean data, with an average value of 1.08 Gb per sample, were analyzed for the presence of single nucleotide polymorphisms (SNPs). Well‐resolved phylogenies of the *P. odoratum* cultivars are constructed; the nonmonophyletic relationship in the plastome‐based phylogenetic trees, yet monophyletic form in the RAD‐based linkage map suggested possibility of hybrid cultivar for *P. odoratum* “Dazhu” (GDDZ), which was further supported by morphological observations. Quality assessment based on the standards of the Chinese Pharmacopoeia on Polygonati Odorati Rhizoma (POR) on the four cultivars used in this study recorded that PORs from *P. odoratum* “Zhongzhu” (GDZZ) met the minimum criteria for the acceptance as raw material for medicinal drug production. This study has provided insights on the morphological variations, genetic background, and medicinal qualities of *P. odoratum* cultivars that could be explored for future genetic improvement as well as breeding programs of *P. odoratum* for POR production.

## Graphical abstract

*Polygonatum odoratum* cultivars can be differentiated morphologically. The nonmonophyletic relationship revealed from the phylogenetic analyses based on the plastome sequences and a monophyletic form in the RAD‐based linkage map suggested the possible presence of hybrids in certain cultivars. Polygonati Odorati Rhizoma from the cultivar Zhongzhu (GDZZ) met the criteria in the Chinese Pharmacopoeia as raw material for medicinal drug production.

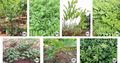

## 1 INTRODUCTION

Over the last decade, there has been an emerging demand for Chinese medicinal herbs due to its popularity across the globe. In order to meet this surge of demand, herbs are now mass‐cultivated in open fields and chambers instead of relying on wild resources (Zhao‐Seiler, 2013). The World Health Organization (WHO) has always been supportive toward the utilization of native medicine (World Health Organization, 2001), with implementing standards in medicinal plant cultivation practice by prioritizing production quality control for medical purposes. This effort resulted to the implementation of good agriculture practice (GAP) certification system for Chinese medicinal farmers, which provides a guide for farmers to grow these herbs as well as a timely revised Chinese Pharmacopeia that archives desired quality and criteria of raw materials that are meant for medical purposes (Zhang et al., 2010). Although the practice of propagating the herb might appear to be simple, yet the inconsistency in the chemical and biological nature expressed in its extracts or plant parts to the desired quality appear to be arguable. Obtaining information on the morphological, genetic background, and phytochemical analysis of these medicinal plant species is a requisite step to deter such inadequacies to aid improvement of their growth performance and yield quality extracts.

*Polygonatum odoratum* (Mill.) Druce, commonly called the Solomon's seal, is a perennial herb from the family of Asparagaceae. It is mainly natural distributed in the East Asia region, including China, Japan, Korea, and Mongolia, and has also been documented in the northern Europe continent and eastern region of Russia. The *P. odoratum* produces fibrous rhizome, which is an essential ingredient in traditional medicine. The dried *P. odoratum's* rhizome, also known as Polygonati Odorati Rhizoma (POR), or Yuzhu (jade bamboo in Chinese), is classified as a medicinal resource with top grade medical and edible properties in ancient medicinal plant records (Liu et al., 2008). It is commonly used as an immune conditioning agent that relieves dryness by quenching thirst and promoting secretion of fluid, promotes blood lipids and glucose reduction, and improves myocardial ischemia. The phytochemical compound‐rich POR includes alkaloids, flavonoids, glycosides, phyto‐hormones, and saponins (Khan et al., 2012; Zhao et al., 2018). It has been recognized by traditional Chinese medicine (TCM) practitioners and is currently archived in the Chinese Pharmacopoeia (Chinese Pharmacopoeia Committee, 2015). The differences in types of polysaccharides in *P. odoratum* when compared to other *Polygonatum* spp. suggested that POR cannot be replaced by other *Polygonatum* spp or closely related rhizomatous herb species due to variation in pharmacological properties (Guo & Tian, 2001). Its public demand as a functional food and TCM for treating diabetes as well as its potency in healing heart diseases and leukemia has led to promoting the cultivation of *P. odoratum* on a large scale in China.

Planting and harvesting of *Polygonatum odoratum* are performed annually during the autumn season. As *P. odoratum* can be propagated sexually (via seeds) and asexually (via rhizome cuttings), the supply for planting material is often sufficient. With the aid of crop breeding techniques, new *P. odoratum* cultivars are introduced by medicinal plant breeders through selection breeding, with the aim to yield high‐quality PORs. Cultivars often use rhizome cuttings to propagate where identical clones can be replicated through asexual means. The morphological and genetic characteristics of these clones should correspond to the main body, in which the desired quality is thought to be conserved. There are two ways to distinguish types of *P. odoratum* that the cultivars exhibit in markets. One is from its morphological characteristics, such as rhizome shape, size, and taste, and the other is by its place of origin. According to the former technique, there have been claims that there are more than 10 different cultivars recorded in literatures that used as POR resources, mainly from the provinces of Jilin, Guangdong, and Hunan (Bi et al., 2016; Ma et al., 2014; Yang et al., 2009, personal observation). The latter categorizes *P. odoratum* into six different types of cultivars, namely Xiangyuzhu (Hunan origin), Haimenyuzhu (Jiangsu origin), Xiyuzhu (Guangdong origin), Dongyuzhu (Zhejiang origin), Guanyuzhu (Northeast China origin), and Jiangbeiyuzhu (Jiangsu and Anhui origins), which all exhibit different phenotypic characteristics. Initially, the major cultivated POR‐producing provinces were confined to Jilin, Guangdong, and Hunan though the high demand in POR has led to the expansion in *P. odoratum* planting sites in neighboring provinces, such as Jiangsu, Zhejiang, Henan, and Liaoning. While there are slight morphological differences between *P. odoratum* cultivars (Chen & Zhou, 2010; Yang, 2004), researches have shown that the harvest and quality of POR may vary across planting regions and types of cultivars (Bi et al., 2016; Bu, 2012; Yang et al., 2009).

Unlike model plants and important crop plants, there is a dearth of published studies in genomics of medicinal plants such as *Polygonatum odoratum*. Genetic information on exploitation of commercial crops such as *P. odoratum* is crucial to lay out a basis of desired traits for the molecular breeding of the crop species. Despite the fact that the selection of cultivar for POR is not greatly influenced by the consumers, farmers would be able to maximize their profit income by selecting their preferred cultivar who is believed to deliver better POR yield and quality. Previous work to characterize different *P. odoratum* cultivars utilized only DNA fingerprinting techniques such as the intersimple sequence repeat (ISSR) markers (Pan et al., 2008), which are dominant in nature. In general, dominant genetic markers are less specific and informative compared with co‐dominant markers. Since band profiles of dominant markers are scored based on presence or absence, the ISSR technique is a multilocus marker that requires the user to amplify a handful of primer sets in order to provide a better resolution in genetic similarity estimates (Ng & Tan, 2015). The misinterpretation during scoring step and lower reproducibility when comparing with codominant markers may result in incorrect genetic evaluation of the targeted specimens (Agrawal & Shrivastava, 2014). Thus, a DNA sequence‐based method is critically needed for the characterization of *P. odoratum* cultivars. Owing to the rapid development in DNA sequencing techniques, the next‐generation sequencing (NGS) technology warrants an opportunity to generate genome‐scale information to provide better resolution in genetic diversity and genome evolution analyses at a much lower cost. The plastid genome (plastome) is reported to be highly conserved in its gene contents and displays a maternal inheritance among angiosperms, thus being useful in phylogenetic studies, population genetics, phylogeography, and species characterization. Mutation hotspots identified in the plastome due to highly variable regions when comparing across several plastomes of closely related species could be developed for phylogeny and species identification at intraspecies or lower taxa levels. On the other hand, the assembly of the complete nuclear genome is a tedious work; thus, researchers have introduced a substitute method via restriction site‐associated DNA (RAD) sequencing technique to provide genome‐scale information of the nuclear genome. The RAD markers are short DNA fragments adjacent to the selected restricted enzyme recognition site orders (Baird et al., 2008). The technique is versatile to nonmodel and understudied plant species, in which it could provide thousands of genome‐scale single nucleotide polymorphism (SNP) sites that are beneficial for construction of phylogenetic trees and marker‐assisted selection (Cui et al., 2018). The genome sequences encompass essential information such as plant origin, evolution, development, physiology, inheritable traits, epigenomic regulation, etc., which are basis in molecular breeding of high‐yielding medicinal cultivars and molecular farming of transgenic medicinal strains (Hao & Xiao, 2015).

In this study, we conducted assessments to identify the morphological and vegetative phase differences between the four major *Polygonatum odoratum* cultivars collected from POR farms in Guangdong, Hunan, and Jilin provinces. We also sequenced and characterized the plastomes of each cultivar to construct the phylogenetic tree to identify potential cultivar‐specific barcodes to tell apart the cultivars. A RAD‐based linkage map of these four *P. odoratum* cultivars was constructed to reveal its phylogenetic relationships at the nuclear genome level. Based on the requirements of the Chinese Pharmacopoeia, quality assessment was also carried out on the POR samples derived from the four *P. odoratum* cultivars to determine their suitability as raw material for medicine production. Findings from this study will be beneficial to explore molecular breeding of superior cultivars of the *P. odoratum* in the future.

## 2 MATERIALS AND METHODS

### 2.1 Plant materials

**FIGURE 1:**
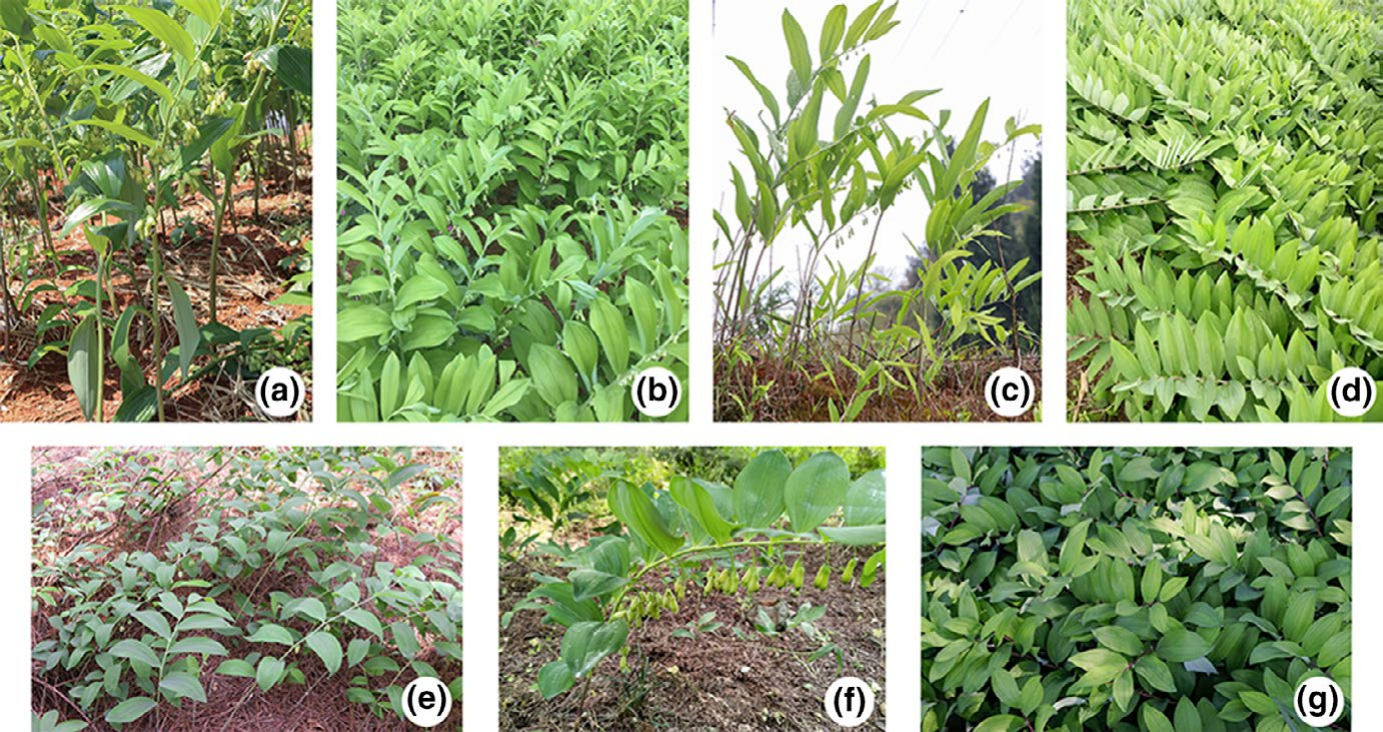
*Polygonatum odoratum* cultivars and *Polygonatum* spp. used in this study. (a) Dazhu (GDDZ), (b) Zhongzhu (GDZZ), (c) Xiangyuzhu (HNXY), (d) Guanyuzhu (JLGY), (e) Guangdong wild accession (GDWA), (f) *P. cyrtonema*, (g) *P. humile*

Sample was collected between March and June 2019 when the plants started to shoot and flower. A total of 18 *Polygonatum odoratum* accessions, consisting of four different cultivars namely Dazhu (GDDZ; *n* = 6), Zhongzhu (GDZZ; *n* = 6) from Guangdong province, Xiangyuzhu (HNXY; *n* = 6) from Hunan province, and Guanyuzhu (JLGY; *n* = 6) from Jilin province, were collected for this study (Table 1, Figure 1). Three additional wild accessions from Guangdong province (GDWA) were collected as outgroups. The samples were dug out together with its soil and transported immediately to the greenhouse in Sun Yat‐sen University for morphology observations. Fresh leaf samples were collected from the plants, kept in aluminum ziplock bags, and stored in −20°C prior to the DNA extraction.

**TABLE 1:** Information on the *Polygonatum odoratum* accessions and other closely related species collected in this study

Species (sample number)	Cultivar	Origin/Collection site	Number of individuals	Collector/collection number	GenBank accession number		
					Complete plastome	nuclear RAD‐seq	ITS
*Polygonatum odoratum*	Dazhu (GDDZ)	Lianzhou, Guangdong	6	Lee, Zhang and Pan; LSY1001 & Chen and Liu; CJ‐956	MW248131	SAMN16792671	MW243055
	Zhongzhu (GDZZ)	Lianzhou, Guangdong	6	Lee, Zhang and Pan; LSY1002 & Chen and Liu; CJ‐957	MW248132	SAMN16792673	MW243056
	Xiangyuzhu (HNXY)	Shaoyang & Shaodong, Hunan	6	Lee, Zhang and Pan; LSY1003 & LSY 1,004	MW248133	SAMN16792674	MW243057
	Guanyuzhu (JLGY)	Dunhua, Huadian & Shulan, Jilin	6	Lee and Chen; LSY1006, LSY1007 & LSY1008	MW248134	SAMN16792675	MW243058, MW243059
	Wild (GDWA)	Lianzhou, Guangdong	3	Lee, Zhang and Pan; LSY1005	MW248130	SAMN16792672	MW243054
*Polygonatum cyrtonema*		Institute of Medicinal Plant Development, Beijing	3	Chen and Lee; CZH629	MW248135	SAMN16792676	–
*Polygonatum humile*		Institute of Botany, Chinese Academy of Science, Beijing	1	BJ‐1‐4001	MN218691	SAMN16792677	MW243060
*Disporopsis fuscopicta*		Xinyi, Guangdong	1	Lee and Chen; LSY1006	MW248136	–	–

### 2.2 Morphological and vegetative observations

Six accession from each cultivar were used for morphological analysis. The vegetative parts, such as stem height, number of leaves, and leaf size, were photographed and documented. For the crop emergence, flowering and fruiting period of each cultivars were based on the observation and interviews with the farmers that were carried out during the sample collection. A total of 12 large‐scale plantation sites, four sites per province, were visited to obtain a comprehensive record on their flowering and fruiting period.

### 2.3 Molecular studies

#### 2.3.1 DNA extraction

A total of 10 mg of fresh leaf samples from each accession were pulverized into powder form using mortar and pestle, with the aid of liquid nitrogen. Genomic DNA was extracted using DN15‐Plant DNA Mini Kit (Aidlab Biotechnologies, China) following the manufacturer's protocol. In order to extract sufficient DNA concentration, each accession was extracted with at least two replicates which were pooled together after the DNA extraction process for quantification and sequencing. The quality and quantity of the DNA product were determined using NanoDrop™ (Bio‐Rad, USA), and DNA samples were kept in −20°C freezer storage prior to sequencing of the plastome and nuclear RAD data.

#### 2.3.2 Plastome sequencing, assembly, and annotation

For the plastome construction of *Polygonatum odoratum* cultivars, one accession was selected as standard for each cultivar. A genomic library with an insert size of ~350 bp was prepared using a TruSeq DNA Sample Prep Kit (Illumina, USA) and sequenced on an Illumina NovaSeq platform (Illumina, USA) for pair‐ends of 150 bp at Guangzhou Jierui Biotechnology Company Ltd (Guangdong, China). Adapter sequences were then removed using NGS Toolkit (Patel and Jain 2012) and raw reads were initially de novo assembled using NOVOPlasty 2.7.2 (Dierckxsens et al. 2017), with the *rbc*L gene sequence of *P. odoratum* (GenBank accession: KC704957) acting as the seed sequence. The assembled plastome was initially annotated using the online annotation tool GeSeq (Tillich et al., 2017) and then manually checked for errors. The final annotated plastome was visualized by drawing a circular plastome map with OGDRAW (Greiner et al., 2019).

#### 2.3.3 Plastome comparison, nucleotide variability, and selection pressure

The GC content was analyzed using Genomics %G~C Content Calculator (https://www.sciencebuddies.org/science‐fair‐projects/references/genomics‐g‐c‐content‐calculator). The plastome sequences of the five *Polygonatum odoratum* accessions were aligned using MAFFT (Katoh et al., 2019), and the resultant alignment was calculated for its variable and parsimonious sites using DnaSP (Rozas et al., 2017). The pairwise distances between the different accessions were calculated using MEGA7 (Kumar et al., 2016), based on Kimura 2‐parameter (K2P) model under uniform rates, by replicating 1,000 bootstraps. Gaps and missing data were treated as pairwise deletion. Nucleotide variability among the four *P. odoratum* cultivars (GDDZ, GDZZ, HNXY, and JLGY) was visualized under mVISTA program (Frazer et al., 2004), using GDWA as the reference. A sliding window analysis was conducted to detect nucleotide variability (Pi) in the plastome across the five accessions using DnaSP v5.1 (Librado and Rozas 2009) based on a window length of 100 bp and a step size of 50 bp.

#### 2.3.4 RAD library preparation and sequencing

For RAD sequencing, a double‐digestion method was applied. Six accessions were selected as representatives for each cultivar, while three accessions—each one for the *Polygonatum odoratum* wild samples, *P. cyrtonema*, and *P. humile*. A total of 100 ng of genomic DNA were extracted from each accession and digested using 5 U each of two restriction enzymes, namely *MluCI* and *SphI* (New England Biolabs, USA) at 37°C for 5 hr. The restriction–ligation reactions were heat‐inactivated at 65°C for 20 min with T4 DNA ligase (New England Biolabs, USA), ATP (New England Biolabs, USA), *MluCI* adapter, and *SphI* adapter. Subsequently, the mixture was incubated at 12°C for 12 hr. A 40‐µl preamplification reaction was conducted using 5 µl of the diluted restriction–ligation mixture using a pair of single‐selective‐nucleotide primers (*EcoRI* and *MseI* adapters). PCR amplification was conducted on a T100™ thermal cycler (Bio‐Rad, USA) with thermal settings programmed as follows: 20 cycles of 94°C for 30 s, 56°C for 60 s, and 72°C for 60 s. Prior to the PCR purification step, PCR products were pooled and incubated at 37°C with *MluCI*, *SphI*, T4 DNA ligase, ATP, and *EcoRI*+MseI adapters (Illumina, USA). The pooled samples were further purified using a E.Z.N.A.® Cycle Pure Kit (Omega Bio‐Tek, USA) and separated on a 2% agarose gel via electrophoresis. Paired‐end sequencing was performed on purified fragments extracted from the agarose gel ranging from 350 to 500 bp (with indexes and adaptors) in size via Illumina NovaSeq platform (Illumina, USA).

#### 2.3.5 SNP calling

Prior to phylogenetic tree analyses, quality filtering and loci assembly were performed using Stacks v1.40 (Catchen et al., 2013) under default parameters. SNP loci that were found present in more than 12 accessions and with allele frequency greater than 0.05 were included for subsequent analysis. To reduce the inclusion of undesirable sequences, RAD tags with depth above 1,000 were excluded. SNP identification was carried out in the alignment results using populations, in which alleles with a minimum occurrence of 0.6 times were regarded as true polymorphisms.

#### 2.3.6 Phylogenetic analyses

Phylogenetic tree was constructed based on the plastome sequences of five *Polygonatum odoratum* accessions (GDDZ, GDWA, GDZZ, HNXY, and JLGY), and seven other *Polygonatum* species (*P. cirrhifolium*, *P. cyrtonema*, *P. humile*, *P. kingianum*, *P. sibricum*, *P. stenophyllum*, *P. verticillatum*) that were downloaded from the GenBank. Plastome sequence alignment was carried out using MAFFT (Katoh et al., 2019), while three closely related taxa, *Asparagus officinalis* (KY364194), *Disporopsis fuscopicta* (MW248136), and *Heteropolygonatum ginfushanicum* (MW363694) were included as outgroup. For RAD‐seq dataset, the vcf file was converted to fasta format using TASSEL v5.0 (Bradbury et al., 2007) prior to ML and BI analyses. Phylogenetic analyses were conducted using RAxML (Stamatakis, 2014) and MrBayes (Ronquist et al., 2012) available in the CIPRESS Science Gateway web portal (Miller et al., 2010) for both the plastome and RAD‐seq datasets. The maximum likelihood (ML) tree was constructed using the general time‐reversible (GTR) with discrete gamma distribution (+G) (=GTR+G) nucleotide substitution model, and 1,000 bootstrap replicates were applied on each branch node. The Bayesian inference (BI) tree was constructed based on default parameters, with minor adjustments: a mixed substation type (Nst) was selected for the likelihood model and 2,000,000 generations were determined for the Markov chain Monte Carlo (MCMC), with data sampling collected every 100 generations. The final tree files from both analyses were visualized under FigTree v1.4 (Rambaut, 2012). The resultant trees constructed using the RAD‐seq dataset were rooted with *P. cyrtonema* and *P. humile* was included as outgroup.

### 2.4 Pharmacopeia standard analyses

Assessments were based on the standard provided by the Chinese Pharmacopeia (Chinese Pharmacopoeia Committee, 2015) on POR as a raw material for TCM. For the subsequent analyses, PORs that were at least three years old were selected for the study. Four different cultivars were sampled for subsequent analyses. As for the water content analysis, six accessions from each cultivar were selected and examined in our own laboratory in Sun Yat‐sen University, where three accessions that were selected from each cultivar were sent to Guangdong Institute of Analysis of the China National Analytical Center in Guangzhou for the remaining analyses.

#### 2.4.1 Water content

Since POR are sold in its dried form, fresh POR have to be dried prior serving. To obtain the average water content present in each cultivar, fresh POR samples were sliced horizontally with a thickness of 5 mm and weight of 5 g in total. Oven and petri dishes were preheated at 100–105°C prior placing the POR samples in petri dishes to be heated at 105°C for 5 hr. Afterward, specimens were let to cool down for 30 min prior weighing. The heating process is repeated but only for 1 hr and reweighed until the weight of the POR sample is reduced to 5 mg consecutively. Based on the weight difference between the fresh and dried POR samples, the percentage of water content of the cultivar was determined.

#### 2.4.2 Total ash content

To ascertain the total content of ash, dried POR samples were grinded into powder form with a blender that would be able to be sieved using a No. 2 sieve. A total of 3 g sample were placed into an incinerator and slowly heated until they were carbonized; then, the temperature was subsequently increased to 600°C and finally stopped when the samples were fully incinerated. Based on the weight difference between the pretreated and treated samples, the percentage of the total ash content was determined and which should not exceed 3.0%.

#### 2.4.3 Ethanol‐soluble cold extract content

A total of 4 g powder sample were diluted into 100 ml of 70% ethanol solution in a 300‐ml sealed conical flask by constantly shaking for 6 hr and thereafter left to sit for 18 hr. The dilution was immediately dry‐filtered to obtain a filtrate of 20 ml. The filtrate was transferred into a steam evaporator to be treated with steam and then oven‐dried at 105°C for 3 hr. The sample was left to cool down for 30 m in the oven where its weight was recorded. Based on the weight of the dried samples, the percentage of ethanol‐soluble extract was determined and should be more than 50%.

#### 2.4.4 Polysaccharide quantification assay

A total of 1 g powder sample were diluted with 100 ml of water in a beaker. The mixture was reflux‐heated for 1 hr before filtered through absorbent cotton. The process was replicated to obtain two amounts of filtrates. The filtrates were then combined and transferred into a 100‐ml measuring flask where water was added until it reached a final volume of 100 ml, which was afterward shaken prior isolating 2‐ml into 10 ml of ethanol solution. Thence, the mixture was stirred and centrifuged. Upon extraction, the precipitant was dissolved in water and transferred into a 50‐ml measuring flask where the solution was topped‐up with water until it reached the final volume of 50 ml. Subsequently, 2 ml of the solution was pipetted and added into 1 ml of 4% phenol solution prior to quantification using light spectrophotometer. Using glucose as the reference, the polysaccharide content in the POR sample should be more than 6%.

## 3 RESULTS

### 3.1 Morphological characteristics and vegetative stages of *Polygonatum odoratum* cultivars

The four *Polygonatum odoratum* cultivars shared similar morphological features, such as having alternate leaves, terete rhizomes, and inflorescences with 1–4 flowers. However, there were minor differences that differentiated the cultivars, whereby GDDZ had longer stem size that was recorded at a height of between 0.82 and 1.02 m and at the same time it produced a greater average number of leaves, that is, 20 (Table 2). On the other hand, JLGY was recorded to have the smallest stem size. The morphological features between GDZZ and HNXY are identical, with distinct morphological difference in which the leaf size for HNXY was at least one size larger than GDZZ. Vegetative phases were similar for all four cultivars, in which the time between the crop emergence and fruiting stages was three months for GDZZ and HNXY, but four months for GDDZ and JLGY. At the starting of spring season, crops for GDZZ and HNXY were recorded to sprout initially in March, followed by GDDZ in April, and finally JLGY in May.

**TABLE 2:** Information on the morphological features and vegetative phases of four different *Polygonatum odoratum* cultivars used in this study

	GDDZ	GDZZ	HNXY	JLGY
Stem height (m)	0.82–0.92–1.02	0.55–0.59–0.63	0.53–0.58–0.61	0.46–0.49–0.53
Number of leaves	18–20–22	8–9–11	7–8–10	7–8–9
Leaf size (cm ×cm)	11.4–12.0 × 4.6–4.9	6.3–6.6 × 3.4–5.0	13.3–13.8 × 6.3–7.2	10.0–10.6 × 4.8–5.5
Crop emergence	April	March	March	May
Flowering season	June	April	April	July
Fruiting season	July	May	May	August

Note

Cultivar names: GDDZ, Dazhu; GDZZ, Zhongzhu; HNXY, Xiangyuzhu; JLGY, Guanyuzhu.

### 3.2 Plastome features of *Polygonatum odoratum*

**FIGURE 2:**
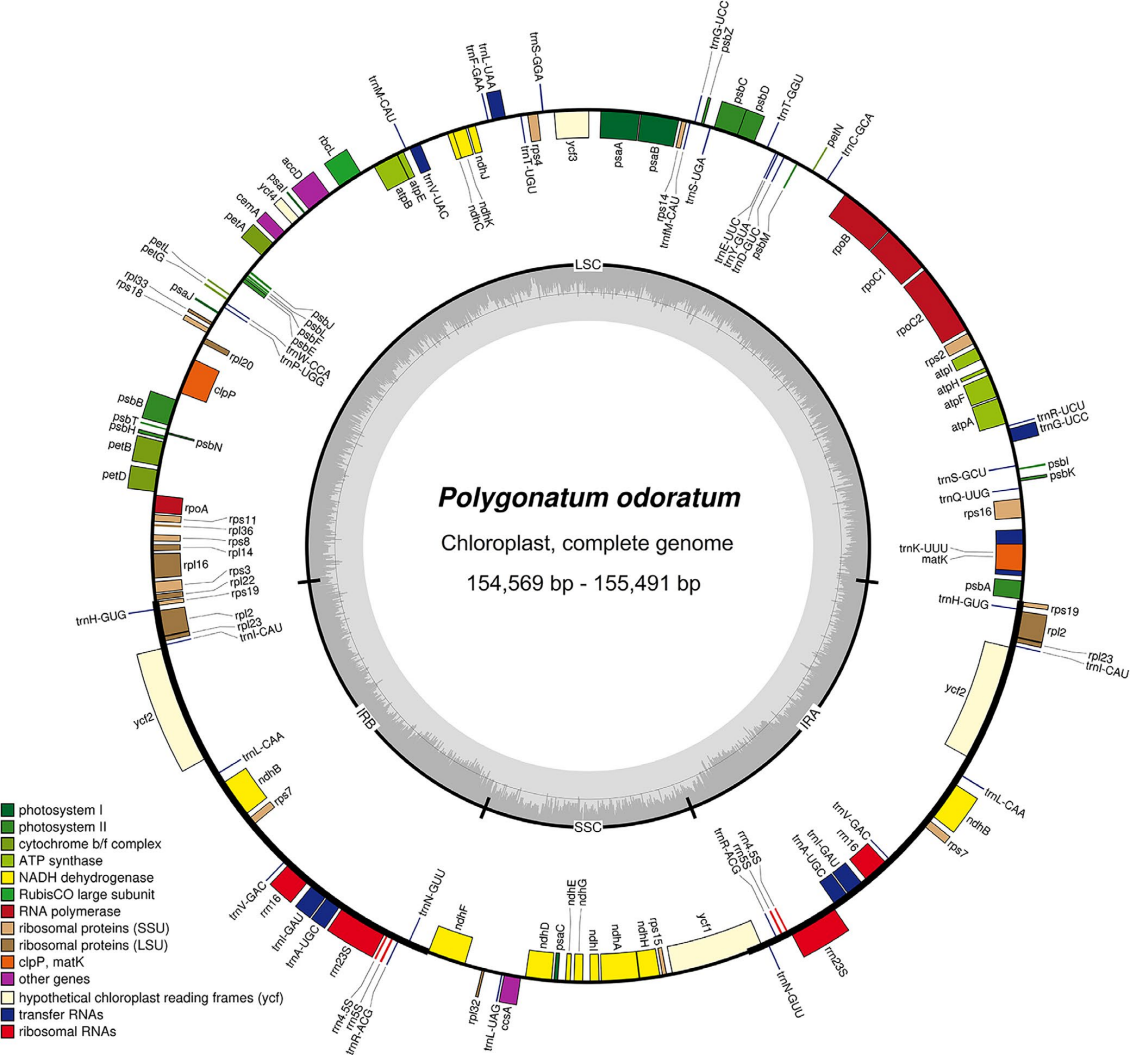
Plastome map of *Polygonatum odoratum*. Genes are color‐coded according to their different functions. Genes inside the circle are transcribed clockwise, while genes outside the circle are transcribed counterclockwise

The overall plastome sizes of *Polygonatum odoratum* ranged from 154,569 bp (GDWA and GDZZ) to 155,491 bp (GDDZ) (Figure 2). All five plastomes exhibited typical quadripartite structure regions that consisted of a large single copy (LSC; 83,486–84,459 bp) and a small single copy (SSC; 18,292–18,471 bp), separated by a pair of inverted repeats (IRs; 26,302–26,370 bp). All five plastomes contained equal number of genes, which was 131 genes, including 85 protein‐coding (CDS) genes, 38 transfer RNA (tRNA) genes, and 8 ribosomal RNA (rRNA) genes. Among these genes, 18 genes were duplicated in both IRs, including six CDS genes, eight tRNA genes, and four rRNA genes. The complete plastome sequences were deposited in the NCBI GenBank database and catalogued under the accession numbers MW248130‐MW248134.

### 3.3 Plastome variations

**FIGURE 3:**
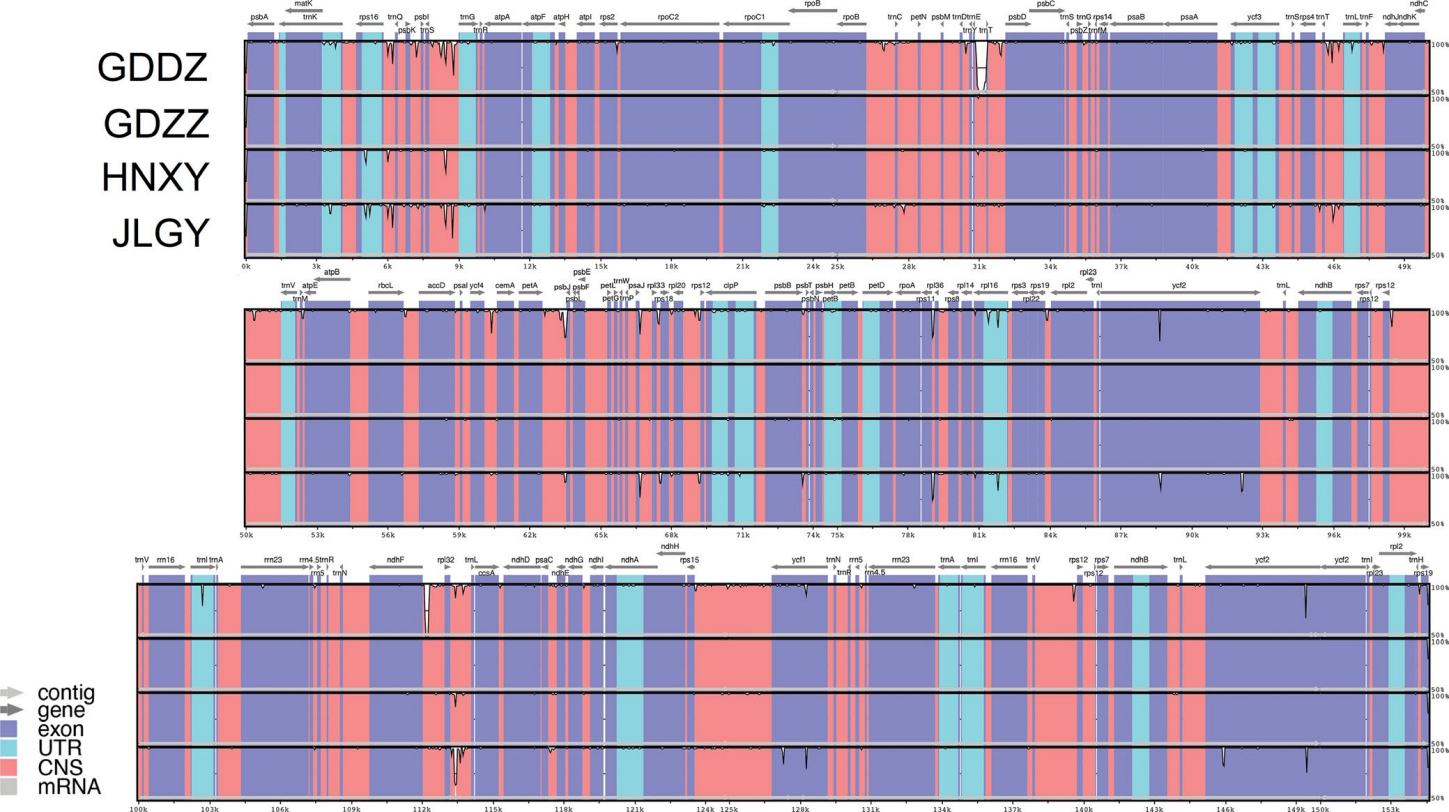
Plastome comparison of four *Polygonatum odoratum* cultivars using mVISTA under Shuffle‐LAGAN mode. Figure legend describes the direction and types of gene regions using color codes. Probability threshold was set at 50%, and the plastome of *P. odoratum* from the wild (GDWA) was selected as the reference genome

**FIGURE 4:**
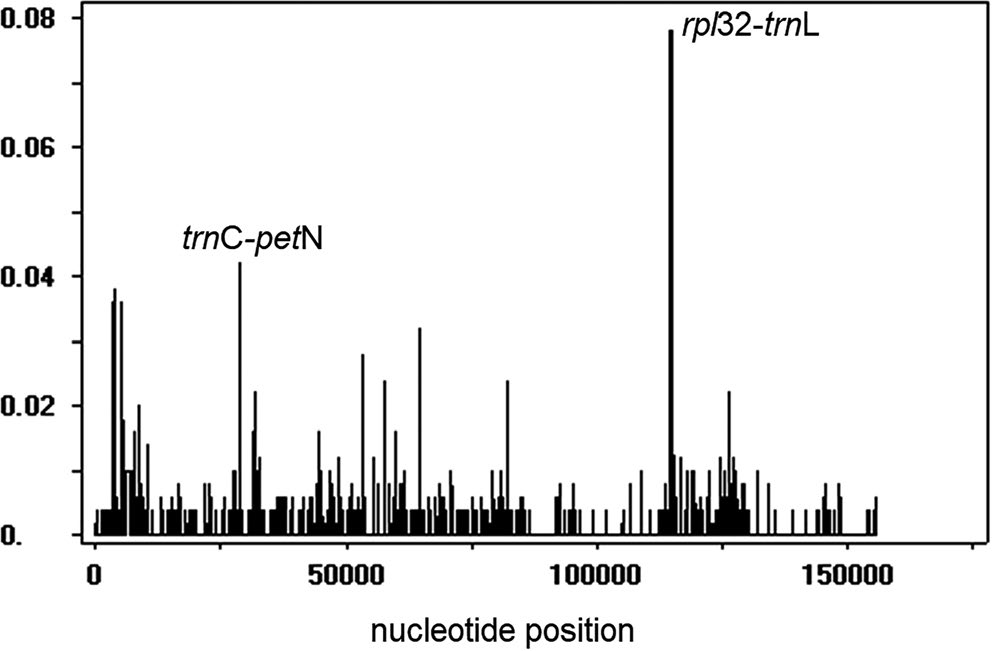
Identification of highly variable sites in the plastome sequences of four different *Polygonatum odoratum* cultivars

The GC contents for plastome of the five *Polygonatum odoratum* accessions were 37.7% for GDDZ and JLGY, and 37.8% for GDWA, GDZZ, and HNXY. The alignment of the complete plastome sequences of the five *P. odoratum* accessions resulted in a 155,987‐bp long sequence, containing 337 singleton variable sites, in which 335 were with two variants and two were three variants. The singletons were abundant in IRA (*n* = 213), followed by the SSC region (*n* = 69), LSC region (*n* = 34), and IRB (*n* = 21). A total of 106 parsimony informative sites were detected, in which 79 were detected in the LSC region and 29 were located in the SSC region. Both the IR regions were detected in every three sites. The smallest pairwise distance was detected between GDWA and GDZZ (0.000), while the largest pairwise distance was detected between GDDZ and JLGY (0.0022) (Table 3). Plastome comparison via mVISTA displayed high similarity in nucleotide variability among the five *P. odoratum* accession. When compared to the reference genome, the plastome of GDDZ exhibited a distinct gap in the intergenic spacer region *trn*E‐*trn*T of the LSC region that was further identified as a unique insertion of a 11‐bp repeat motif (Figure 3). Based on the sliding window analysis, the average nucleotide diversity (Pi) was 0.00129. With the Pi value cutoff point set at 0.04, two highly variable regions were detected at the aligned sequence region at 28,552 bp to 28,701 bp and 114,586 bp to 114,787 bp, which were the intergenic spacer regions, *trn*C‐*pet*N and *rpl*32‐*trn*L (Figure 4).

**TABLE 3:** Pairwise distance of the complete plastome sequences between the wild *Polygonatum odoratum* accession and four different cultivars used in this study

	GDWA	GDDZ	GDZZ	HNXY
GDDZ	0.0021			
GDZZ	0.0000	0.0021		
HNXY	0.0004	0.0021	0.0003	
JLGY	0.0012	0.0022	0.0013	0.0014

Note

Cultivar names: GDDZ, Dazhu; GDZZ, Zhongzhu; GDWA, wild accession; HNXY, Xiangyuzhu; JLGY, Guanyuzhu.

### 3.4 RAD tag generation and SNP genotyping

This study used 31 accessions and generated 37.51 Gb of raw data that produced 250,068,346 paired‐end reads (Table 4). The raw data were deposited in NCBI SRA database under the accession number PRJNA678294. A total of 33.64 Gb clean data were obtained postfiltering, resulting in an average of 1.08 Gb (1,085,141,327 bp) clean reads per accession. The average effective rate, Q20, Q30 in the five accessions were 99.97, 98.52, and 96.75, respectively. Upon loci clustering, a total of 4,661,457 loci were obtained but only 317 loci, containing 45,494 sites, succeeded the sample constraint test. Upon genotyping, 4,189 variable sites were identified, with a mean genotyped site of 143.28 bp per locus.

**TABLE 4:** Information on the nuclear RAD data obtained in this study

Sample	Number of Raw reads	Number of RAD tags	Filtered reads	Clean reads	Digestion reads	Digestion ration (%)	Read depth
GDDZ_1	3,759,548	446,413	173	3,312,962	1,869,275	62.6	13.69
GDDZ_2	6,035,698	354,579	180	5,680,939	3,187,325	59.7	17.25
GDDZ_3	4,714,898	800,674	274	3,913,950	1,930,696	61.4	9.29
GDDZ_4	8,179,260	750,738	5,045	7,423,477	4,775,925	71.5	47.17
GDDZ_5	4,821,630	608,250	2,765	4,210,615	2,591,967	71.5	45.25
GDDZ_6	5,465,824	637,002	3,159	4,825,663	2,949,639	70.4	45.14
GDWA_1	4,809,502	825,492	532	3,983,478	1,920,445	60.2	10.26
GDWA_2	4,554,856	734,897	453	3,819,506	187,427	60.2	11.12
GDWA_3	6,678,064	171,045	211	6,506,808	3,914,090	61.8	16.92
GDZZ_1	4,134,030	1,263,723	685	2,869,622	888,093	51.3	17.04
GDZZ_2	7,363,588	2,242,822	1,280	5,119,486	1,409,442	46.4	12.40
GDZZ_3	7,783,038	3,468,209	1,058	4,313,771	1,186,333	45.3	13.10
GDZZ_4	6,069,732	816,350	3,544	5,249,838	3,358,826	75.5	45.94
GDZZ_5	9,123,018	851,815	5,597	8,265,606	5,071,896	68.4	48.10
GDZZ_6	5,194,738	816,815	2,806	4,375,117	2,695,933	74.6	45.24
HNXY_1	8,723,048	160,891	274	8,561,883	5,028,909	59.8	18.49
HNXY_2	8,568,614	222,593	243	8,345,778	4,995,569	61.4	17.77
HNXY_3	12,763,076	307,174	404	12,455,498	8,185,584	67.3	16.50
HNXY_4	5,844,404	858,968	414	4,985,022	2,552,844	61.5	8.88
HNXY_5	5,274,736	257,311	184	5,017,241	3,034,820	63.7	16.36
HNXY_6	11,264,224	316,593	328	10,947,303	6,483,048	60.9	18.66
JLGY_1	9,462,054	270,648	2	9,191,404	6,130,992	68.7	9.72
JLGY_2	4,313,896	335,189	6	3,978,701	2,492,760	68.3	9.58
JLGY_3	8,067,190	230,784	5	7,836,401	5,613,163	73.8	9.28
JLGY_4	3,447,418	141,789	2	3,305,627	2,303,117	72.7	9.03
JLGY_5	11,449,168	166,151	5	11,283,012	7,267,619	65.3	11.98
JLGY_6	20,463,346	438,871	5	20,024,470	13,808,259	70.5	9.85
*P. cyrtonema*_1	10,025,482	1,545,802	5,337	8,474,343	5,058,388	72.7	48.06
*P. cyrtonema*_2	9,240,188	2,095,664	4,307	7,140,217	3,508,751	68.5	41.91
*P. cyrtonema*_3	4,930,654	813,229	2,687	4,114,738	2,610,954	78.9	48.52
*P. humile*	21,436,882	1,732,128	6,065	19,698,689	9,984,306	54.1	62.33

### 3.5 Phylogenetic inferences

**FIGURE 5:**
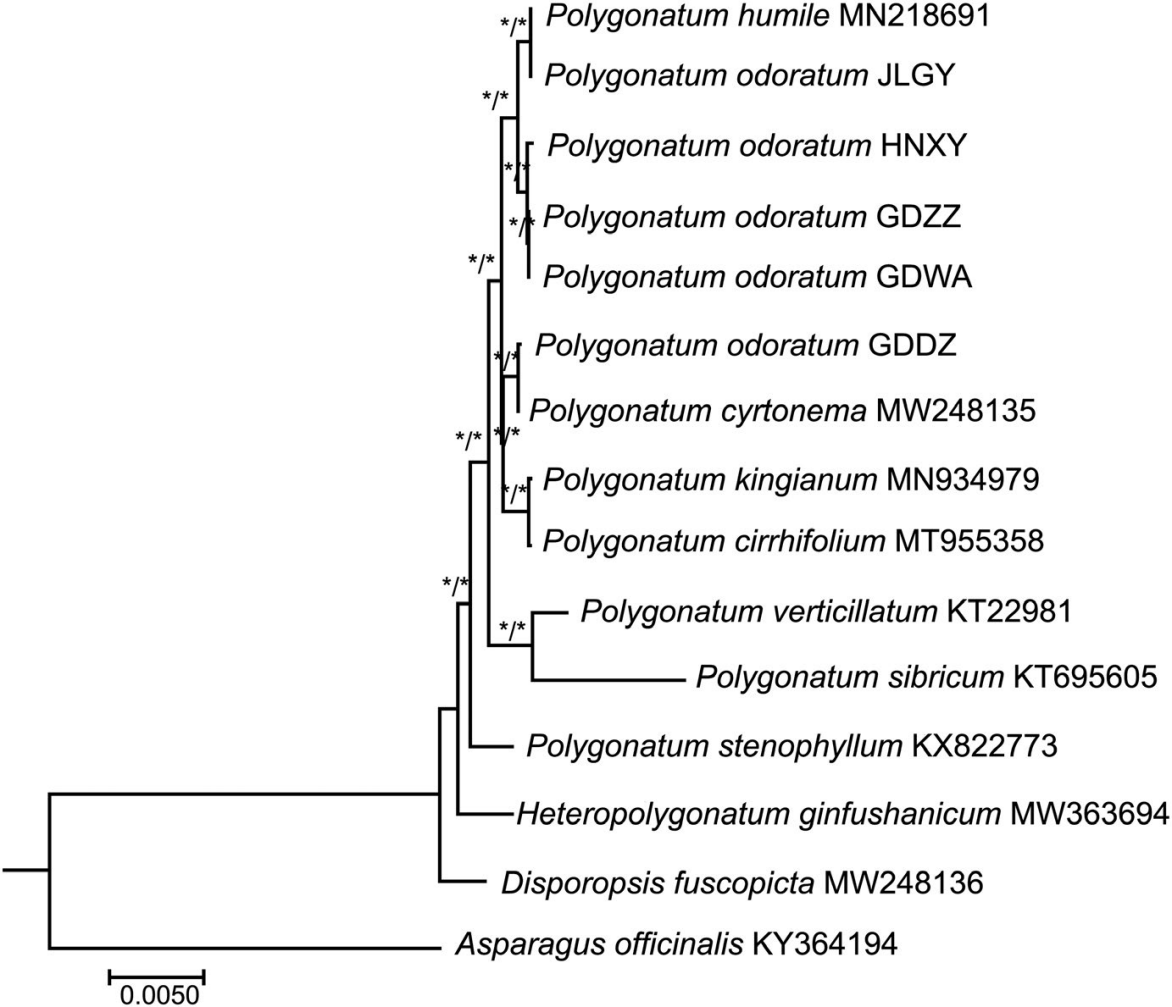
Phylogenetic analyses of 11 taxa from *Polygonatum* based on the complete plastome sequences. The asterisk symbol indicates that strong bootstrap support (BS) value or posterior probability (PP) was recorded at the branch node, in which BS ≥ 75% for the maximum likelihood (ML) tree and PP ≥ 0.90 for the Bayesian inference (BI) tree. Two closely related species, *Asparagus officinalis* (KY364194) and *Disporopsis fuscopicta* (MW248136), were included as outgroups

*Polygonatum odoratum* was not recovered as monophyletic in our analyses of the complete plastomes, and two groups were recovered with strong branch supports in both the ML and BI trees (Figure 5). GDDZ formed a clade with *P. cyrtonema* and was sister to the *P. kingianum* + *P. cirrhifolium* clade, while GDZZ, GDWA, and HNXY were clustered together, and JLGY was sister to *P. humile*.

**FIGURE 6:**
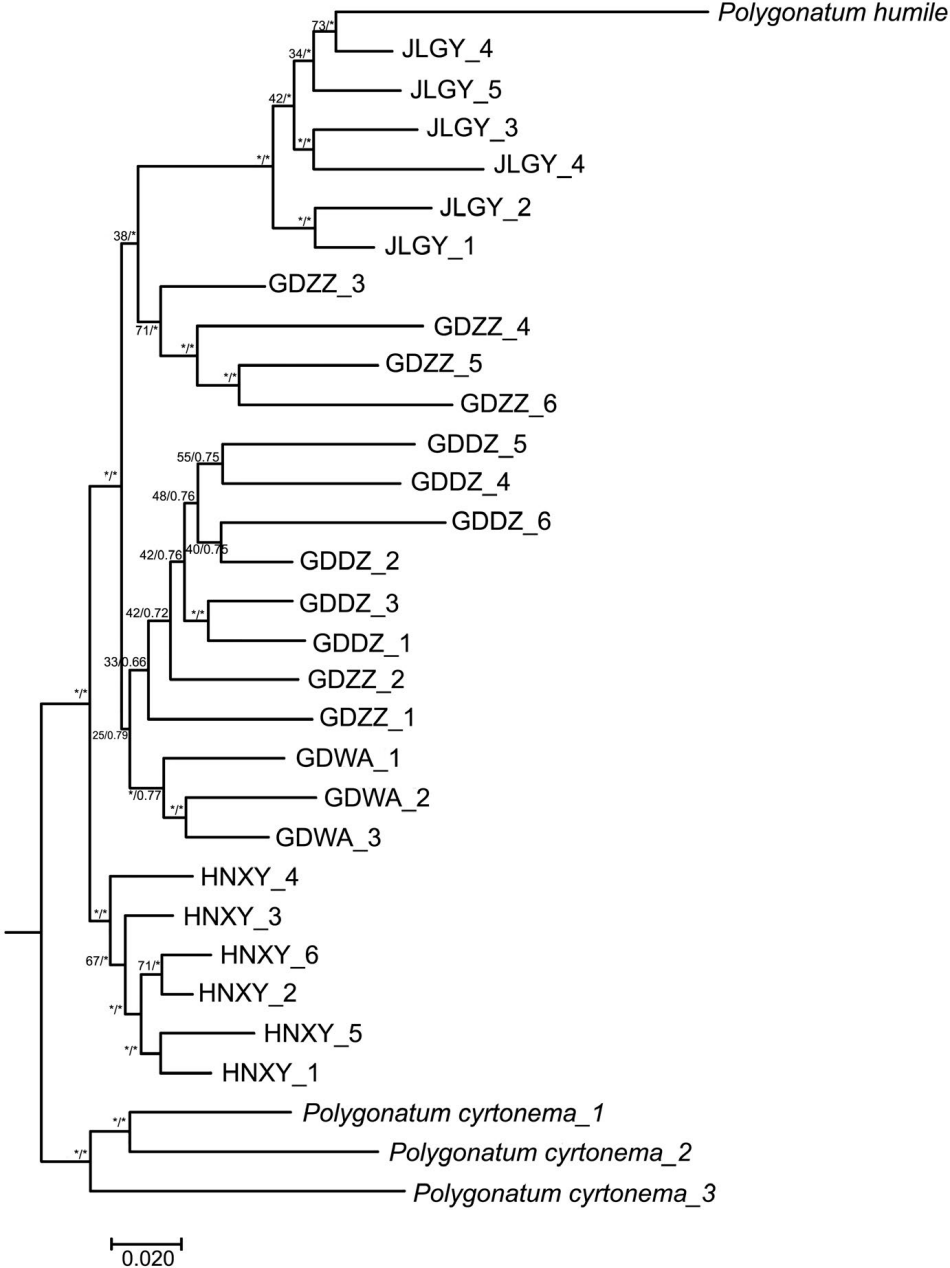
Single nucleotide polymorphism (SNP)‐based RAD linkage map of five different *Polygonatum odoratum* accessions used in this study. The asterisk symbol indicates that strong bootstrap support (BS) value or posterior probability (PP) was recorded at the branch node, in which BS ≥ 75% for the maximum likelihood (ML) method and PP ≥ 0.90 for the Bayesian inference (BI) method. *Polygonatum cyrtonema* was placed at the root, while *Polygonatum humile* was included as outgroup

A 9,024‐bp long aligned SNP dataset was obtained for genetic subdivision and phylogeny reconstruction. The SNP‐based ML tree displayed three distinct clades for the five *Polygonatum odoratum* accessions under strong bootstrap supports (BS ≥ 75%) in which three GDWA were clustered with all six GDDZ and two GDZZ (GDZZ_1 and GDZZ_2); four GDZZ (GDZZ_3, GDZZ_4, GDZZ_5, and GDZZ_6) were clustered with all six JLGY; HNXY formed an independent clade (Figure 6). On the contrary, the BI tree revealed a stronger backbone for the phylogenetic tree, in which four distinct clades were observed for the five *P. odoratum* accessions under strong Bayesian probability posterior (PP ≥0.90). The GDZZ clade that consisted of four GDZZ samples (GDZZ_3, GDZZ_4, GDZZ_5, and GDZZ_6) were well resolved and separated from the JLGY clade. *Polygonatum humile* was found to be sister to JLGY in both the ML and BI trees.

### 3.6 Qualification of POR

For fresh POR samples, JLGZ was recorded with the highest percentage in water content, followed by GDDZ, while both the GDZZ and HNXY recorded the lowest water content percentage (Table 5). According to the monographs from the Chinese Pharmacopoeia (Chinese Pharmacopoeia Committee, 2015), POR samples for all four accessions complied with the percentage of water content allowed in the dried samples and the percentage of ethanol‐soluble extract, in which the former should not exceed 16% and the latter should not be less than 50%. However, irregularities were recorded for JLGZ POR samples for the percentage of total ash content as well as GDDZ and HNXY POR samples for percentage of polysaccharide content. The requirements for POR were not more than 3% for the percentage of total ash content, while not less than 6% for the percentage of polysaccharide content.

**TABLE 5:** Qualification test of Polygonati Odorati Rhizoma (POR) derived from four different *Polygonatum odoratum* cultivars used in this study based on the standard provided by the Chinese Pharmacopeia for use as raw material in Chinese traditional medicine

	Water content (%)		Total ash content (%)	Ethanol‐soluble extract (%)	Polysaccharide content (%)
	Fresh sample	Dried sample			
GDDZ	0.73	0.07*	2.4*	52.9*	4.4
GDZZ	0.67	0.07*	1.8*	62.5*	6.4*
HNXY	0.67	0.06*	1.5*	76.7*	4.1
JLGZ	0.77	0.00*	6.0	50.3*	13.9*

Note

Cultivar names: GDDZ, Dazhu; GDZZ, Zhongzhu; HNXY, Xiangyuzhu; JLGY, Guanyuzhu

* Indicates that the readings comply with the minimum requirements provided by the Chinese Pharmacopeia (2015) on POR.

## 4 DISCUSSION

### 4.1 Morphological diversity in *Polygonatum odoratum* cultivars

We observed that the *Polygonatum odoratum* cultivars had similar morphological features but can still be differentiated when carefully compared. We found out that the distinct morphological characteristics that could differentiate these cultivars were their stem sizes and leaf size. Although the size of the rhizome differs between cultivars, we anticipated that the size of the rhizome could be influenced by the plant spacing (Tiwari et al., 2019); thus, rhizome size is not a useful morphological trait to differentiate the cultivars. Generally, the number of segments on the rhizome is due to the age of the plant (Ohara et al., 2007), while the planting periods are often three years for plant materials from rhizome cuttings and five years for plant materials from seeds in order to obtain an optimum production yield.

### 4.2 Potential hybrids in *Polygonatum odoratum* cultivars

The structure of the plastome for *Polygonatum odoratum* was well conserved across the five accessions used in this study. The five plastomes obtained from this study did not show any general differences in gene content. Intraspecific plastid sequence variability was very low despite sampling the accessions from different cultivars across regions. In contrast, genetic distances were higher between GDDZ and the other four accessions ranging from 0.0021–0.0022; whereas genetic distance between GDZZ and GDWA was 0.000, suggesting that the two accessions could be sharing similar maternal inheritance. Based on the plastome sequence‐based ML and BI tree, *P. odoratum* was not monophyletic. Given the nonexistence of large‐scale plastome sequencing data in *P. odoratum* cultivars, we hypothesized that there was a possibility that the genetic material for GDZZ and HNXY were identical based on the high similarity in plastome sequences, verified by the high similarity in morphological features and vegetative phases. This hypothesis was accepted when GDZZ, GDWA, and HNXY were clustered together in the plastome‐based ML and BI trees under strong bootstrap supports. However, in the SNP‐based ML and BI trees, the HNXY clade branched out under a strong bootstrap support, which separated this cultivar from the rest. This suggested that the evolution of nuclear genome HNXY could be independent and there might be an absence of gene flow between HNXY and other accessions.

On the contrary, the sistership between GDDZ and *Polygonatum cyrtonema* in the plastome‐based phylogenetic trees suggested that the maternal genetic material for GDDZ had a closer relationship with *P. cyrtonema* than *P. odoratum*. While there was resemblance between GDZZ and *P. cyrtonema* in regards to large stem size, the number of inflorescences and rhizome shapes were not. GDDZ was 2–4‐flowered and came with terete‐shaped rhizomes whereas *P. cyrtonema* was 2–7‐flowered and had moniliform rhizomes. We note that the basic chromosome numbers for *P. cyrtonema* and *P. odoratum* are different, in which *P. cyrtonema* is *x* = 9, or 11 and *P. odoratum* is *x* = 8, 9, 10, or 11 (Chen & Zhou, 2005; Zhao et al., 2014). Generally, hybridization involving progenitors with two different basic chromosome numbers will lead to sterile hybrids (Shokrpour, 2019). The hypothesis that GDDZ could potentially be a hybrid of *P. cyrtonema* and *P. odoratum* was further inclined when we were informed by the farmers that GDDZ was propagated through rhizome cuttings. However, the small sampling size in this study may give a false indication regarding the presence of hybrid, based solely on the results of molecular experiments. Therefore, additional experiments to validate these claims on GDDZ are necessary to provide useful information for further genetic studies. All six GDDZ were categorized within the same clade along with the GDWA accession and a fraction of GDZZ. Based on the molecular placement of GDDZ in both the plastome‐based and SNP‐based, we speculate that GDDZ could be a hybrid cultivar between *P. cyrtonema* and the wild *P. odoratum* accession from Guangdong. Even though GDZZ could strike off as a potential genetic material for GDDZ, we disregarded the probability because in the province of Guangdong, GDDZ are often grown adjacent to GDZZ within the same locale. Despite the rare and late pollination occurrence of GDZZ with the early blooming of GDDZ in the same field, there might be occurrence of gene flow. Since GDZZ and JLGY were from different regions, thence we were not able to postulate possibilities of gene flow between these two cultivars although the two cultivars were clustered under the same clade in the SNP‐based ML tree but were well resolved in the BI tree. The strong sistership between JLGY and *P. humile* revealed from the plastome‐based and the SNP‐based phylogenetic trees suggested that they could be closely related to each other, while both taxa shared identical morphological characteristics—*P. humile* is distinguishable from *P. odoratum* by having hispidulous leaves abaxially and *P. humile* is typically 1‐flowered compared with *P. odoratum* which is generally 1–4‐flowered, but sometimes could be 7‐flowered in cultivated individuals (Chen & Tamura, 2000). Furthermore, *P. humile* naturally habitats in the Northeast region of China, where JLGY is largely cultivated. It was difficult to specify *Polygonatum* species that hold the closest genetic relationship to GDDZ and JLGY because of the dearth of studies on plastome and nuclear data for members of *Polygonatum*. Nevertheless, characterization of other members under the genus *Polygonatum* could justify the phylogenetic relationship of these cultivars.

**FIGURE 7:**
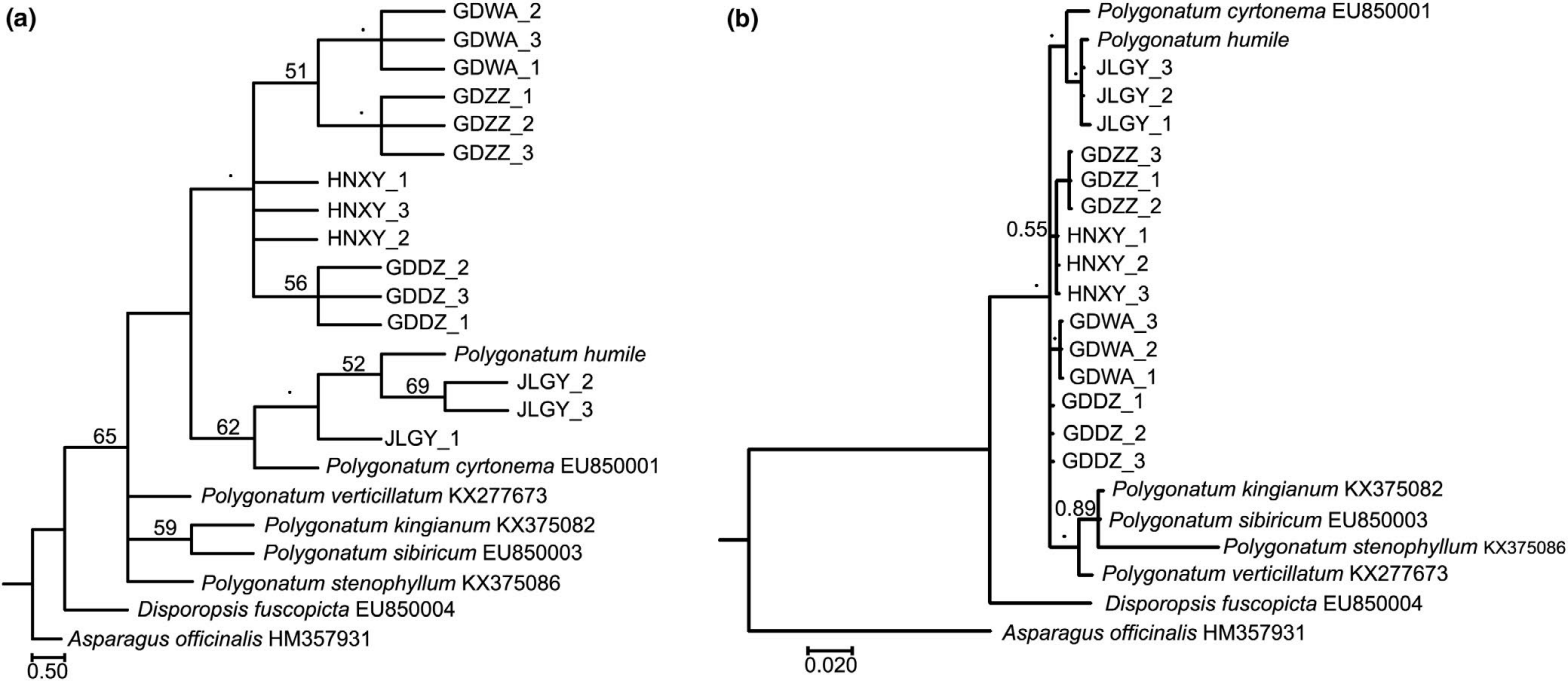
Phylogenetic analyses of five different *Polygonatum odoratum* accessions based on the internal transcribed spacer (ITS) sequences using (a) maximum likelihood (ML) method and (b) Bayesian inference (BI) method. The asterisk symbol indicates that strong bootstrap support (BS) value or posterior probability (PP) were recorded at the branch node, in which BS ≥75% and PP ≥0.90

**FIGURE 8:**
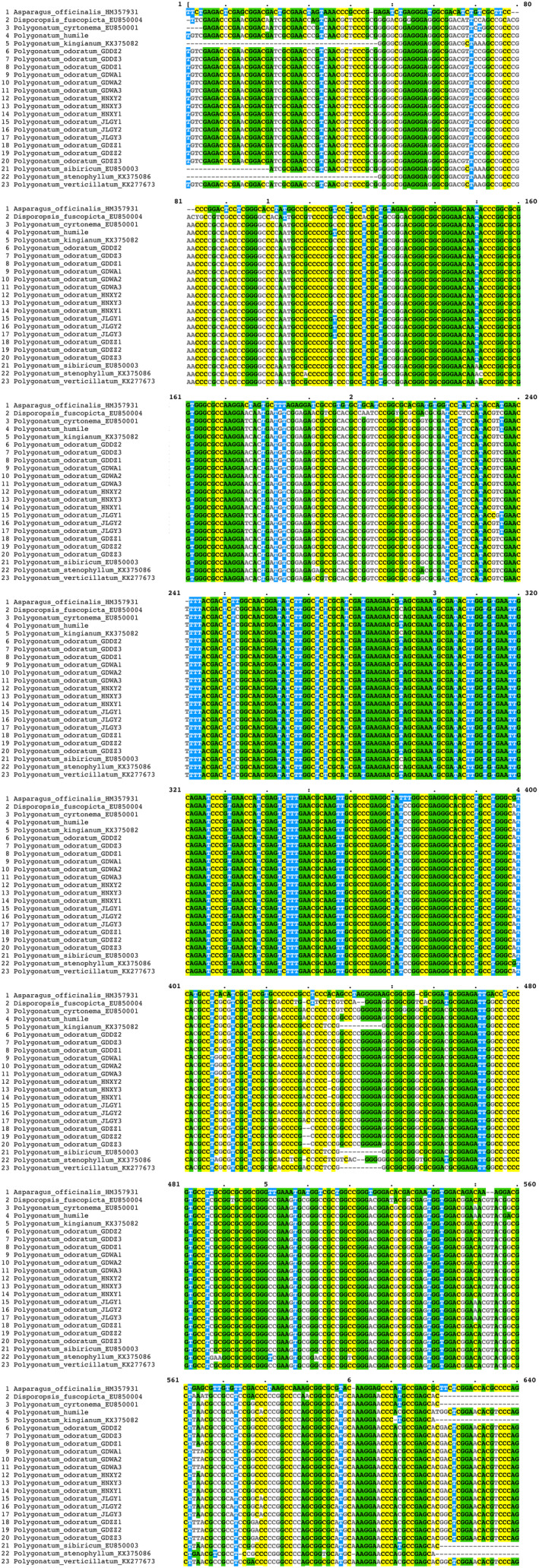
Alignment of the internal transcribed spacer (ITS) sequences of 21 taxa of *Polygonatum* representing seven *Polygonatum* species, including four different *P. odoratum* cultivars, prior to phylogenetic reconstruction. The sequence alignment was trimmed with trimAl (Capella‐Gutiérrez et al., 2009) and two species, *Asparagus officinalis* (HM357931) and *Disporopsis fuscopicta* (EU850004) were included as outgroup

Albeit the nuclear ribosomal DNA internal transcribed spacer (ITS) sequences were proven to have the strength in distinguishing closely related species and identifying the genetic source of hybrids and cultivars for economic crops (Hidayat et al., 2013; Yonemori et al., 2002; Yoon et al., 2009), the case was different in *Polygonatum*. An attempt to construct the ITS‐based phylogenetic trees for *Polygonatum odoratum* accessions in this study resulted in both the ML and BI tree displaying weak backbone structure (Figure 7). Although a handful of SNPs were present in the sequence alignment (Figure 8), the ITS sequence was not powerful enough to resolve the phylogenetic relationships within *P. odoratum*. Consequently, similar events were reported for other genera in the family Asparagaceae, that is, *Asparagus* (Fukuda et al., 2012), and *Ophiopogon* (Wang et al., 2014), while the combined data of the plastid intergenic spacer region, *pet*A‐*psb*J, and ITS sequences are only capable to recognize at best the three distinct sections within *Polygonatum* (Floden & Schilling, 2018).

In this study, the identity of the SNP loci by SNP calling was not reported as several attempts were reverted with intergenic spacer regions and also largely due to a reference genome on *Polygonatum* was unavailable at the moment. By means of the reference genome from closely related species, *Asparagus officinalis*, failed to provide sufficient information in this study, while RAD tags for *A. officinalis* containing SNPs that complemented to *Polygonatum odoratum* were found to be very limited (data not shown). Therefore, the proposal to obtain a draft genome for *P. odoratum* could certainly aid in the gene identification of desired traits. The estimated genome size for *P. odoratum* was recorded to be 9,613.73 Mbp (Siljak‐Yakovlev et al., 2010), which will undeniably require an enormous account of resource and budget to sequence the full nuclear genome. Hence, it was suggested that the desirable genome coverage when performing analysis with RAD‐seq data should be based on its sequencing rigor in order to reveal sufficient informative sites (Feng et al., 2018); the average raw sequence data for *P. odoratum* samples yielded 9.262 Mbp (Table 4). Despite the low genome coverage rate, the SNP‐based phylogenetic trees were able to provide better species resolution when compared to the ITS‐based phylogenetic trees which reported higher bootstrap supports along the backbone of the SNP‐based phylogenetic tree.

### 4.3 POR production for TCM resource

*Polygonatum odoratum* is a well‐known traditional medicinal plant recognized for its potential genes in regulating both the polysaccharide and isoflavonoid biosyntheses that produce polysaccharides and isoflavonoids for pharmaceutical purpose (Zhang et al., 2020), as they are as natural chemicals in nutrition for general well‐being (Liu et al., 2015; Miadoková, 2009). In China, the quality standard criteria of raw materials for herbal products are defined in the Chinese Pharmacopoeia, which includes prior testing for characterization and standardization of raw materials before being produced as a medicinal drug. In our study, only the GDZZ POR samples met the minimum criteria of the quality standards set by the Chinese Pharmacopoeia. It is a common phenomenon that distinct spatiotemporal variations in phytochemical profiles in medicinal plants exist (Dhami & Mishra, 2015). Nonetheless, it is important to note that the Pharmacopoeia's standards are designated for quality control of POR solely for medicinal use or herbal drug productions. Although POR contains a small amount of toxicity (Liu et al., 2008), it is still considered to be safe and can be consumed as a supplement (Chau & Wu, 2006). The Chinese community has a strong belief in the effectiveness of herbal diet to prevent sickness and diseases (Chen & Yang, 2008; Luo et al., 2019). POR are often purchased and consumed as an ingredient in the preparation of herbal soup as well as in tea and wine making (Liu & Liu, 2015). At present, cultivated PORs are being sold in the market based on their region of origins. For example, cultivated POR from Jilin and Liaoning provinces are retailed in Japan and Korea, while cultivated POR from Hunan province is usually exported to other foreign countries except Japan and Korea; whereas cultivated POR from Guangdong and other provinces are sold locally for domestic consumption (S. Lin, personal communication). Although there are records of selected *P. odoratum* cultivars that excel in both growth performance and yield quality when compared to other *P. odoratum* cultivars and wild *P. odoratum* accessions (Lu et al., 2014; Yang et al., 2009), there is no distinct preference among the consumers in this regard. These cultivars are usually being promoted to farmers as “quality breeds” for POR production due to its capability to yield greater rhizome sizes as well as meeting all the criteria stated in the Chinese Pharmacopeia as medicinal source. Interestingly, there are plant materials which are sold at a much higher price that claim to be “quality breeds” or wildings that contain high medicinal values, but their origins were not disclosed, either due to the herb being a sham or for personal gains through fraudulent business schemes.

## 5 CONCLUSION

In this study, we reported on the morphological variations and differences in vegetative phases of four major *Polygonatum odoratum* cultivars in China. From the aspect of genetics, we sequenced novel complete plastome sequences of these *P. odoratum* cultivars and investigated their phylogenetic relationships at genome‐scale level for both plastid and nuclear inheritance. As for the medicinal aspect, we assessed the medicinal qualities of the PORs derived from these cultivars based on the criteria described in the Chinese Pharmacopoeia. Outcomes of this study revealed that these cultivars can be differentiated morphologically; the polyphyletic placement of *P. odoratum* based on the maternally inherited complete plastome sequence, but monophyletic through the biparentally inherited nuclear SNP‐based RAD linkage map suggested the possible presence of hybrids among the cultivars; *P. odoratum* cultivars with PORs that met the criteria described in the Chinese Pharmacopoeia can be considered for selection of sustainable POR production in TCM application. As a valuable medicinal herb that is popular in the Chinese society and nearby regions, these findings would be helpful for future molecular breeding and selection of superior cultivars for POR production in China.

## CONFLICT OF INTEREST

The authors have no conflicts of interest to declare.

## AUTHOR CONTRIBUTIONS

**Shiou Yih Lee:** Conceptualization (equal); Data curation (equal); Formal analysis (lead); Investigation (lead); Methodology (lead); Writing‐original draft (lead); Writing‐review & editing (equal). **Zhihui Chen:** Data curation (equal); Formal analysis (supporting); Investigation (supporting); Methodology (supporting); Writing‐original draft (supporting). **Zhiming Chen:** Data curation (equal); Investigation (supporting); Resources (supporting). **Jingrui Chen:** Data curation (equal); Formal analysis (supporting); Investigation (supporting); Methodology (supporting). **Xinjian Zhang:** Data curation (equal); Formal analysis (supporting); Investigation (supporting); Methodology (supporting); Writing‐original draft (supporting). **Jiawen Pan:** Data curation (equal); Investigation (supporting); Project administration (lead). **Qiang Fan:** Conceptualization (equal); Funding acquisition (equal); Resources (supporting); Supervision (supporting); Writing‐review & editing (equal). **Wenbo Liao:** Conceptualization (equal); Funding acquisition (equal); Resources (lead); Supervision (lead); Writing‐review & editing (equal).

## Data Availability

Sequence data are available in the GenBank and SRA under accession number MW248130–MW248136, WM234054–WM243060, and SAMN16792671–SAMN16792677 as well as the DRYAD database (accession number: https://doi.org/10.5061/dryad.ncjsxkstz).
